# Is all hypoglycaemia treated as equal? An observational study of how the type of diabetes and treatment prescribed prior to admission influences quality of treatment of inpatient hypoglycaemia

**DOI:** 10.1007/s00592-016-0940-3

**Published:** 2016-11-28

**Authors:** Gregory C. Jones, Jansher Khan, Christopher A. R. Sainsbury

**Affiliations:** Diabetes Department, Gartnavel General Hospital, Glasgow, G11 0YN UK

**Keywords:** Diabetes, Inpatient, Hypoglycaemia, Insulin, Sulphonylurea, Quality of care

## Abstract

**Aims:**

Inpatient hypoglycaemia is common and associated with adverse outcomes. There is often increased vigilance of hypoglycaemia in inpatients with type 1 diabetes (T1DM) compared to type 2 diabetes (T2DM). We aimed to investigate this apparent discrepancy, utilising the time to repeat (TTR) capillary blood glucose (CBG) measurement as a surrogate for engagement with guidelines stating that CBG should be rechecked following intervention within 15 min of an initial CBG of <4 mmol/L.

**Methods:**

This is an observational study of inpatient CBG data from 8 hospitals over a 7-year period. A national diabetes registry allowed identification of individual’s diagnosis and diabetes therapy. For each initial (index) CBG, the TTR for individuals with T2DM—on insulin or sulphonylurea—was compared with the TTR for individuals with T1DM, using a *t* test for significance performed on log(TTR). The median TTR was plotted for each group per index CBG.

**Results:**

In total, 1480,335 CBG measurements were obtained. A total of 26,664 were <4 mmol/L. The TTR in T2DM individuals on sulphonylurea was significantly greater than in T1DM individuals where index CBG was ≥2.3 mmol/L (except index CBG 2.6 mmol/L). For T2DM patients receiving insulin significance exists for index CBGs of ≥3.2 mmol/L.

**Conclusions:**

This analysis suggests that quality of care of hypoglycaemia varies according to diagnosis and medication. The group with the highest TTR (T2DM sulphonylurea treated) are possibly the clinical group in whom hypoglycaemia is most concerning. These data therefore suggest a need for education and raising awareness within the inpatient nursing staff.

## Background and aims

Hypoglycaemia is an important co-morbidity in most patients with type 1 diabetes and many with type 2 diabetes and has potentially fatal consequences [1]. Fewer than 20% of patients with type 1 diabetes (T1DM) are free of hypoglycaemia in any year [2]. In patients with type 2 diabetes (T2DM), it has been reported that by 9 months of follow-up 7% of patients on recently initiated insulin or sulphonylurea treatment will have experienced severe hypoglycaemia (hypoglycaemia needing external assistance) [3]. Occurrence of severe hypoglycaemia has demonstrated to be associated with macrovascular events, adverse clinical outcomes and mortality in people with T1DM and T2DM [4, 5]. As well as poor clinical outcomes hypoglycaemia is a complication greatly feared by patients and associated with significant psychological and social burdens [6, 7].

In hospitalised patients with diabetes, hypoglycaemia is common with a reported frequency of between 3.3 and 5.7% [8–11]. In the UK National Diabetes Inpatient Audit, hypoglycaemia occurred in 45.3% of inpatients with T1DM and 31.8% T2DM [12].

Sulphonylureas (SUs) pose a significant hypoglycaemic risk with a reported incidence of 19% of inpatients treated with SUs [13] and one-third of hypoglycaemic episodes attributed exclusively to SU therapy in an audit of 11 acute UK NHS trusts [14]. Although severity of hypoglycaemia was significantly greater with insulin therapy, the number of episodes of hypoglycaemia experienced was similar [13, 14].

Frequency and severity of hypoglycaemia have been associated with an increase in pre- and postdischarge mortality and length of admission [15–17]. Even in patients without diabetes, hypoglycaemia on hospital admission has been linked with a significant increase in inpatient mortality and bed occupancy [18, 19].

Patients experiencing hypoglycaemia (blood glucose < 4 mmol/l) require prompt action with administration of rapid acting carbohydrate or glucagon followed by assessment of response to treatment by repeat blood glucose measurement. The Joint British Diabetes Societies inpatient care guidelines for the treatment of hypoglycaemia recommend that following treatment of hypoglycaemia capillary blood glucose (CBG) is repeated at between 10 and 15 min to ensure successful treatment [12]. This timescale for repeating CBG is also recommended by the American Diabetes Association for all episodes of hypoglycaemia occurring in patients with diabetes [20].

Compliance with guidelines for repeat testing following identification of hypoglycaemia is substandard. In a 5-year analysis of 8 acute hospitals in the UK, it was revealed that following recorded hypoglycaemia events 4.4% of patients had no repeat CBG. Of the repeated measurements, less than 10% had a TTR < 15 min and the median TTR was 80 min. As would seem instinctive, a proportional relationship was seen with TTR and severity of initial CBG values [21].

It has been our experience that in an inpatient setting there is often a high degree of awareness of hypoglycaemia in individuals with T1DM, but less awareness for those with T2DM.

We aimed to investigate for both the presence and scale of this apparent discrepancy in clinical vigilance by utilising the time to repeat (TTR) capillary blood glucose (CBG) measurement as a surrogate for engagement with extant clinical guidelines for hypoglycaemia—which in our institution state that CBG should be rechecked following intervention within 15 min where an initial CBG of <4 mmol/L is identified.

## Patients and methods

Inpatient CBG data were collected from 8 hospitals, comprising a variety of acute and general medical and surgical wards in district general and teaching hospitals, in the Greater Glasgow and Clyde Health Board over a period of 7 years to January 2016. CBG value, time of test, date of test and corresponding patient identifier were extracted from analysis of the Abbott Precision Webb system (Abbott, UK). Episodes of hypoglycaemia were identified as CBG of <4 mmol/l. Repeat CBG testing for the same patient identifier was then identified, and the time between the test was calculated to give the TTR. By merging the dataset with a national diabetes registry (Scottish Care Information Diabetes Collaboration System), it was possible to cross-reference patient identifiers and identify the individual’s diagnosis of T1DM or T2DM.

Primary care prescribing information was available from the registry for all individuals with diabetes. Admissions were deemed to be associated with insulin or SU therapies if a prescription was identified during the 4 months prior to admission. During an admission, the time interval between each CBG measurement was calculated and analysed per initial (index) CBG value. For each index CBG, the TTR for those individuals with T2DM—insulin or SU treated—was compared with the TTR for those individuals with T1DM, using a t test performed on log(TTR) to test significance. The median TTR was plotted for each group per index CBG.

## Results

A summary of results is represented in Table 1. In total, 1,480,335 CBG measurements were obtained by the Abbott system in total. This comprised 406,690 values from 4304 individuals with T1DM, 484,067 values from 5164 individuals with T2DM on insulin therapy and 589,778 values from 13,015 individuals with T2DM on sulphonylureas (SU).

**Table 1:** Summary of diagnosis, therapy, number of CBG values and median TTR (IQR)

Diagnosis and therapy	Number of individuals	Number of CBG values	CBG values <4 mmol/l	Median (IQR) TTR overall (min)	Median (IQR) TTR for CBG 1–3.9 mmol/l (min)
T1DM	4304	406,690	26,664	186 (90–314)	53 (26–112)
T2DM + Insulin	5164	484,067	23,591	305 (159–552)	64 (30–147)
T2DM + SU	13,015	589,778	30,344	355 (198–706)	97 (40–292)

Of these readings, 26,664 were identified as being hypoglycaemic (<4 mmol/L) from individuals with T1DM, 23,591 from individuals with T2DM on insulin and 30,344 from individuals with T2DM on sulphonylurea therapy.

The overall median (IQR) TTR for all index CBG values (1–27.8 mmol/l) was: 186 (90–314) min in individuals with T1DM; 305 (159–552) min in patients with T2DM on insulin; and 355 (198–706) min in T2DM patients on SU.

The median (IQR) TTR for index CBGs in the range of 1–3.9 mmol/L was: 53 (26–112) min in individuals with T1DM; 64 (30–147) min in patients with T2DM on insulin; and 97 (40–292) min in T2DM patients on SU.

**Fig. 1:**
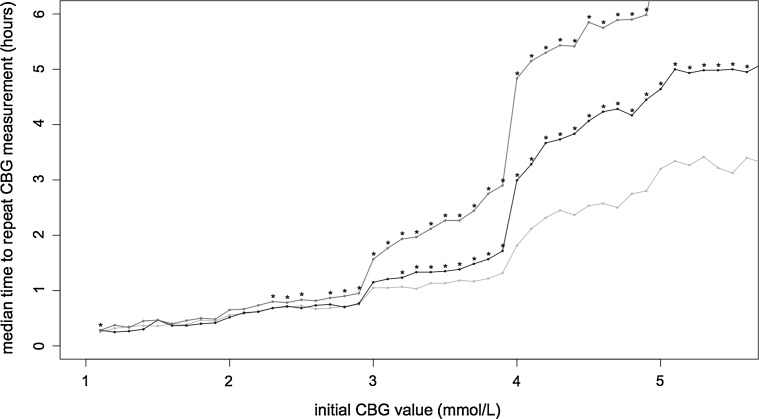
Median time to repeat of CBG by index CBG in mmol/l for T1DM (*green*), insulin-treated T2DM (*black*) and sulphonylurea-treated T2DM (*purple*) (colour figure online)

Figure 1 shows the relationship between the median TTR of CBG compared to index CBG level in patients with T1DM, T2DM treated with insulin and T2DM treated with a sulphonylurea with an indicator of significance where each T2DM group is compared with the T1DM group. The TTR in the subgroup of T2DM individuals on SU therapy is significantly greater than in T1DM individuals where the index CBG is ≥2.3 mmol/L (except index CBG 2.6 mmol/L). For the portion of T2DM patients receiving insulin significance exists for index CBGs of ≥3.2 mmol/L.

## Conclusion

As has been reported previously the nationally agreed standard of repeating CBG following measured hypoglycaemia is not being met in the vast majority of patients [21]. Guidelines suggest identical action for hypoglycaemic CBGs regardless of clinical context. This analysis suggests that the level of adherence to guidance (which is a measure of quality of care) varies according to the underlying diagnosis and prescribed drugs. TTR decreases as the index CBG decreases as clinically expected, and a reduction in TTR is seen at those thresholds where the initial number of the CBG result decreases (e.g. 3.0 vs. 2.9)—as previously reported.

The difference in attitude towards noninsulin therapies was highlighted by the TOPDOC study. Investigation of confidence and approaches to delivery of diabetes care found that postgraduate medical trainees were less likely to alter oral therapy for diabetes management compared to insulin [22].

Rates of hypoglycaemia in T2DM patients on insulin are lower than for T1DM patients, although disparity reduces with advancement of disease [23]. Irrespective of cause hypoglycaemia is associated with a multitude of negative outcomes, and recent publications have identified comorbidity as potentially the most concerning contributor to hypoglycaemia [24], [25]. Patients admitted to hospital are likely to suffer from more advanced disease and comorbidities. They are also more likely to suffer from the risk factors associated with SU-induced hypoglycaemia, namely older age and reduced kidney function [13]. Furthermore, hypoglycaemia and SU therapy have both been implicated in direct cardiotoxicity, although causative evidence in this area is lacking [26].

Thus, although, understandably, there may be increased vigilance of hypoglycaemia in T1DM patients, the group with the highest TTR (T2DM SU treated) are possibly the clinical group in whom the risks associated with hypoglycaemia are greatest.

These data therefore suggest a need for education and raising awareness within the nursing staff within inpatient units. The benefit of such intervention in improving quality of inpatient hypoglycaemia care has been evidenced previously [21].
